# Establishing Predictive Models for Solvatochromic Parameters of Ionic Liquids

**DOI:** 10.3389/fchem.2019.00605

**Published:** 2019-09-03

**Authors:** Vishwesh Venkatraman, Kallidanthiyil Chellappan Lethesh

**Affiliations:** Department of Chemistry, Norwegian University of Science and Technology, Trondheim, Norway

**Keywords:** ionic liquids, machine learning, COSMO-RS, solvatochromic parameters, screening

## Abstract

The use of ionic liquids (ILs) in applications ranging from catalysis to reaction media in organic synthesis has been successfully demonstrated in several cases. For any given IL application, fundamental properties, such as viscosity, thermal stability, and toxicity have to be considered. Another property of interest is the polarity, which is a crucial indicator of solvent effects on chemical processes. Given the near-infinite combinations of cations and anions, experimental determination of solvatochromic parameters, such as the hydrogen-bond acidity and basicity, and dipolarity-polarizability is prohibitive. To address this, we evaluate the utility of alternative schemes based on parameters derived from COSMO-RS (COnductor-like Screening MOdel for Real Solvents) computations. The scheme is applied to a large library of yet-to-be-synthesized ionic liquids, to identify promising candidates for applications in biomass dissolution.

## 1. Introduction

The ability to favorably tune solvent properties through a suitable selection of the constituent ions, a negligible vapor pressure and excellent thermal stability has made ionic liquids (ILs) an attractive alternative to conventional organic solvents. They have been used extensively in many applications—pharmaceuticals (Marrucho et al., 2014), coatings and lubricants (Zhou and Qu, 2017), fuel cells (MacFarlane et al., 2014), catalysis (Dai et al., 2017), and robotics (Chiolerio and Quadrelli, 2017). Of particular interest is the processing of biological macromolecules, such as cellulose, chitin, wool, etc., which are linked by strong H-bonds (Zhang et al., 2017). An important criterion while selecting ILs for a given application is their polarity which describes the global solvation capability of the solvent interest (Weingärtner, 2008; Hallett and Welton, 2009). Polarity is widely defined as the sum of all the intermolecular interactions between the solvent and a solute (Reichardt and Welton, 2010). While interactions leading to the chemical transformation of the solute are excluded, other interactions (Coulombic, directional, inductive, dispersion, hydrogen bonding, electron pair donor and acceptor forces) are used in the determination of solvent polarity (Chiappe and Pieraccini, 2005; Hallett and Welton, 2011). Since different experimental methods highlight different aspects of these interactions, many polarity scales have been proposed that can yield different values for the same solvent (Katritzky et al., 2004; Reichardt and Welton, 2010).

Owing to the inefficiency associated with single parameter polarity scales that are unable to consider the complexity of interactions involved, Kamlet and Taft introduced a multi-parameter polarity scale composed of hydrogen bond acidity (α) (Taft and Kamlet, 1976), hydrogen bond basicity (β) (Kamlet and Taft, 1976; Yokoyama et al., 1976) and dipolarity/polarizability effects (π^*^) (Kamlet et al., 1977). Similar methods have been implemented for the measurement of polarity of ILs (Crowhurst et al., 2003; Coleman et al., 2009; Jeličić et al., 2009; Doherty et al., 2010; Khupse and Kumar, 2010; Teixeira et al., 2010). In order to obtain Kamlet-Taft parameters for a new solvent, considerable experimental efforts need to be invested because these solvent parameters are measured as an average value of many measurements involving a series of probes. The measurement of the parameters for ILs, is in particular difficult as they are very sensitive to the impurities present in them. An additional requirement is that for ease of measurement, the ILs should be colorless and liquid at room temperature, which is not always the case. Owing to the use of different probes (dyes) used in the process, significant discrepancy has been observed between the measurements from different labs.

Given the innumerable possible combinations of anions and cations, it is evident that it would be almost impossible to experimentally determine the polarity parameters for all ILs. While attempts to establish a common polarity scale for ILs have been carried out (Lungwitz and Spange, 2008; Lungwitz et al., 2008, 2010; Wu et al., 2008), accounting for the complex nanostructure and the interactions is challenging and often show contradictory results (Rani et al., 2011; Wang et al., 2019). In order to calculate the polarity for yet to be synthesized ILs, recent research has focused on predictive models (Cláudio et al., 2014; Kurnia et al., 2015). Models based on cation-anion interaction energies [calculated using the COnductor-like Screening MOdel for Real Solvents—COSMO-RS (Klamt, 1995)] have been found to suitably describe multiple interactions and their effect on reactions (Chen et al., 2007; Guo et al., 2007). However, the proposed models are limited to very small datasets and more specifically, to ILs where the anion or cation is held constant. In this article, we extend the COSMO-RS scheme to a larger collection of ILs and examine the efficacy and utility of the proposed models.

## 2. Materials and Methods

### 2.1. Data Collection

For a set of 234 ILs (55 anions and 110 cations), Kamlet–Taft empirical polarity scales for the hydrogen-bond acidity (α), basicity (β), and dipolarity-polarizability (π^*^), with the dye set Reichardt's Dye, N,N-diethyl-4-nitroaniline and 4-nitroaniline were extracted from literature, a primary source being Rani et al. (2011). Table 1 provides a summary of the data. While values for β were more prevalent, similar number of cases were found for α and π^*^. The structures span various cation families: imidazolium, pyridinium, pyrrolidinium, morpholinium, ammonium, phosphonium, sulfonium, and azepanium, while the anions include NTf_2_, BF_4_, dicyanamide, acetate, nitrate, and halides. Almost half of the ILs belong to the imidazolium family (126 ILs), followed by ammonium (27 ILs) and pyrrolidinium (18 ILs). Detailed analysis of the data is presented in the Results section. An Excel file containing the chemical names of the cations and anions, property values for the ILs used in this study and the corresponding references is provided in the Supplementary Material.

**Table 1:** Summary of the experimental data.

**Property**	**Range**	**N_IL_**	**N_cation_**	**N_anion_**
*α*	0.18–1.41	208	107	40
*β*	0.06–1.61	229	105	45
π^*^	0.73–1.85	208	107	40

*The number of ILs, cations, and anions are specified in the last three columns*.

### 2.2. Computational Methods

Starting with structures of the cations and anions (in SMILES format), the three dimensional coordinates were generated using the OpenBabel software (O'Boyle et al., 2011) and further subjected to a conformer search. For each structure, the lowest energy conformation was retained. Each structure was subsequently subjected to density functional theory optimization using the Becke-Perdew (Becke, 1988) (BP86) functional along with the balanced triple valence plus polarization function (def2-TZVP) basis set and the resolution of identity (RI) approximation (Weigend and Häser, 1997; Weigend and Ahlrichs, 2005). In the first step, a gas phase geometry optimization was performed, followed by the COSMO calculation (represented in the COSMO model by a dielectric medium with a infinite dielectric constant). The quantum chemical COSMO computations were carried out using the program ORCA 3.0 (Neese and Wennmohs, 2015). For each cation and anion, the ORCA program outputs a compressed COSMO file with the extension .ccf which are subsequently used for derivation of other values.

### 2.3. COSMO-RS Descriptors

We chose to represent each IL by a vector of numbers calculated as the sum of the terms corresponding to the participating cation and anion (based on an equimolar cation-anion mixture). The energy terms include: the van der Waals interaction energy (*H*_*vdW*_—non-electrostatic contribution representing dispersive interactions), enthalpy due to hydrogen bonding (*H*_*HB*_), electrostatic misfit interaction energy (*H*_*MF*_), mean interaction energy (*H*_*int*_), ring correction (*E*_*ring*_—a small correction for ring structures), dielectric energy (*E*_*diel*_—the free electrostatic energy gained by the solvation process). These values were obtained by using the COSMO files (.ccf) as input to the COSMOTherm (version C3.0) software (Eckert and Klamt, 2015). In order to ensure an electroneutral mixture in the COSMO-RS calculation, the cation and anion have been treated separately and are used in equal amounts. For the chosen functional and basis set, the parametrization set BP_TZVP_C30_01601 was used for the calculations. Scripts were then used to retrieve the values for the aforementioned descriptors from the output files produced by the software. Following Preiss et al. (2010), we also calculated the symmetry number σ to be the geometric mean of the individual symmetry factors of cation (σ_+_) and anion (σ_−_), i.e., $\sigma =\sqrt{\sigma_{+}\;\sigma_{-}}$.

### 2.4. Statistical Analysis

To model the data, multiple linear regression (MLR) and conditional inference tree based (CTREE) analysis were used. For the MLR model, the best subsets regression (Zhang, 2016) approach was used wherein, all possible models using a specified set of predictors are compared. Given the relatively low number of variables, an exhaustive search based on testing all possible combinations of the predictor variables was carried out. The best overall MLR model was then defined as the model that maximizes the adjusted *R*^2^ and minimizes the Bayesian information criterion (Gatu and Kontoghiorghes, 2006). In the CTREE approach (Hothorn et al., 2006), tree-structured recursive partitioning is combined with multiple statistical test procedures (such as permutation tests) for unbiased variable selection and to prevent overfitting. In all cases, the model performance was assessed using the squared correlation (*R*^2^), root mean square error (*RMSE*), and the average absolute relative deviation (*AARD*) given by:

(1) $$\begin{array}{r} AARD\left(\%\right)=100\times \frac{\sum_{i}^{N}|\frac{\hat{y_{i}}-y_{i}}{y_{i}}|}{N} \end{array}$$

where *y*_*i*_ is the experimental value of the property, $\hat{y_{i}}$ the predicted value, and *N* the number of ILs in the data set. The data analysis was carried out using the statistical computing software R (R Core Team, 2019). The R packages *leaps* (Lumley, 2017) and *party* (Hothorn et al., 2006) were used to carry out the best subsets regression and tree-modeling, respectively.

## 3. Results and Discussion

**Figure 1:**
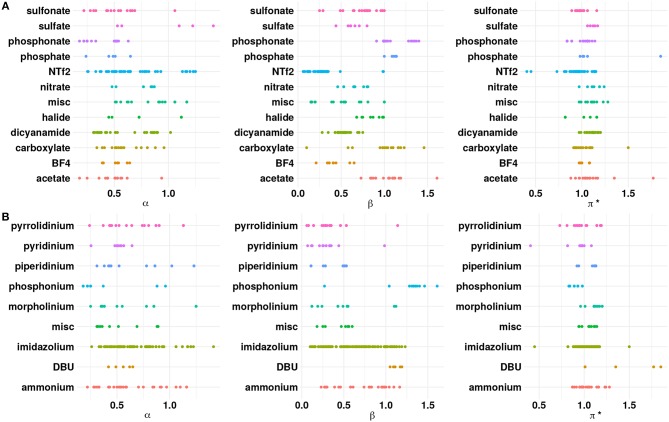
Dot plots of α, β, and π^*^ with respect to the **(A)** anions and **(B)** cations. The “misc” category for anions includes phosphite, perchlorate, tricyanomethanide, thiocyanate, PF_6_, SbF_6_, and dimethoxy(oxo)phosphanuide. The “misc” category for the cations includes pyrazolium, octanium, sulfonium, and azepanium.

As a preliminary step, the ILs were analyzed with respect to the anion chemistries. Figure 1 shows the distribution of the properties with respect to the cation and anion classes present in the available experimental data. Among the cations reported in literature, imidazolium based ILs have relatively higher α values compared to other cations. The substitution of the acidic proton at the C2 position in the imidazolium ring with a methyl/ethyl group, for instance, can reduce α for imidazolium based ILs (Spange et al., 2014). On the other hand, a weakly(strongly) coordinating anion will increase(decrease) the α value. Pyridinium cations possess higher α compared to tetraalkylammonium cations (Spange et al., 2014). This may be due to the highly polarized hydrogen atom near the positively charged nitrogen atom in the pyridine ring. The strong electron withdrawing nature of the pyridine facilitates the formation of the acidic hydrogen atoms. The presence of longer alkyl groups on the cationic core can decrease the α value because of inductive effects and steric hindrance. Phosphonium and sulfonium based ILs exhibit lower α compared to the ammonium ILs because the hydrogen atom (CH) on the phosphonium/sulfonium cationic core are less polarized (Berg, 1978; Shukla and Preetz, 1980). From Figure 1, it is clear that the β values for a majority of the anions are in the range of 0.2–0.8, which indicates that ILs belong to the class of solvents with moderately high hydrogen bond basicities, such as acetonitrile (Chiappe and Pieraccini, 2005; Reichardt, 2005). However, ILs containing anions, such as acetate/carboxylate, chloride and phosphonate have higher β > 1.0. Those with weakly coordinating anions, such as Tf_2_N, PF_6_, BF_4_, and N(CN)_2_ occupy the lower end of the β range. A closer look at Figure 1 shows that α and β values are closely associated, i.e., higher the α, greater the value of β (Spange et al., 2014).

### 3.1. Modeling Results

**Figure 2:**
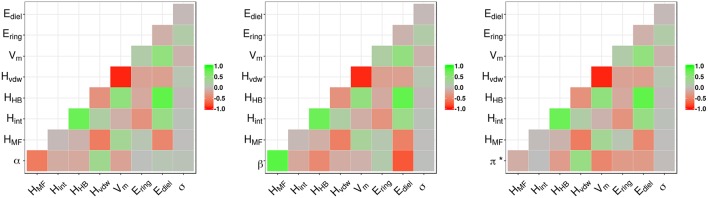
Correlation plots of α, β, and π^*^ with the COSMO-RS calculated parameters.

The correlation plots in Figure 2 for α, β, and π^*^, respectively, explore the relationship between the properties and the COSMO-RS variables. The hydrogen bond acidity (α) is only loosely correlated with most of the variables with the largest values reported for the misfit interaction energy (*H*_*MF*_ − *r* = −0.53) and the van der Waals energy (*H*_*vdw*_ − *r* = 0.36). For π^*^, the correlations with all variables are generally weak with values below 0.50. A summary of the model performance metrics for the three different properties is shown in Table 2. Although results for β are encouraging, the obtained values for α and π^*^ are rather poor. While the non-linear decision tree based approach achieves similar results for β, performances for the other properties offer only a small improvement. For β in particular, a four-parameter model is produced given by:

(2) $$\begin{array}{r} \beta =-0.165+0.135H_{MF}-0.052H_{int}+0.043H_{HB}-2.311V_{m}+\epsilon \end{array}$$

which achieves a relatively high *R*^2^ = 0.87. The results obtained are comparable with those reported by Cláudio et al. (2014) for a smaller dataset. Interestingly, Cláudio et al. (2014) suggested that the basicity was largely seen to depend only on the hydrogen bond strength of the IL. Assessment of the relative variable importance (Lindeman et al., 1980) in the model (shown in Equation 2) reveals that the electrostatic misfit and hydrogen bond interactions dominate the basicity of ILs, with minor contributions from the molar volume and the mean interaction energy.

**Table 2:** Summary statistics for the fitted models.

**Method**	**Property**	**R^2^**	**AARD (%)**	**RMSE**
	*α*	0.42	28.10	0.20
MLR	*β*	0.87	25.61	0.13
	π^*^	0.28	6.84	0.11
	*α*	0.69	20.26	0.18
CTREE	*β*	0.87	23.70	0.15
	π^*^	0.54	5.07	0.11

*Plots of the observed vs. predicted and the computed tree for α are provided in the Supporting Information*.

The earlier study by Kurnia et al. (2015) analyzed the hydrogen bond acidity trends for 17 NTf_2_-based ILs. The authors observed that the hydrogen-bonding energies *H*_*HB*_ play a major role, with contributions from *H*_*MF*_ and *H*_*vdw*_. Our analysis carried out for a larger set of 60 NTf_2_-based ILs suggests a close relationship between *H*_*HB*_ and α as well as π^*^ (see Figure F1 and Equations E1–E6 in the Supporting Information). Somewhat analogous trends for ILs based on dicyanamide for α. However, the computed model for α_*NTf*2_ (see Table T2) only achieves a *R*^2^ = 0.51 compared with the higher value of 0.94 obtained by (Kurnia et al., 2015). This could be attributed to the use of a larger dataset where the structural and chemical variability is considerably broad. For instance, the ILs in our dataset consist of different cationic cores, such as imidazolium, pyrrolidinium, azepanium, piperidinium, pyridinium, tetraalkyl phosphonium and tetraalkyl ammonium, sulfonium (Mellein et al., 2007; Chiappe et al., 2011; Rani et al., 2011; Hauru et al., 2012; Kurnia et al., 2015). In addition to cationic core with simple alkyl groups (C1-C8), cations containing different functional groups, such as nitrile (Lee and Prausnitz, 2010), ether (Chiappe et al., 2011; Rani et al., 2011), silyl (Rani et al., 2011), and sulfinyl groups (Lee and Prausnitz, 2010). One other issue is related to the reported values for hydrogen-bond acidity which do show some strong variations. For example, values of 0.47 (Hauru et al., 2012) and 0.36 (Spange et al., 2014) for 1-butyl-3-methylimidazolium acetate are reported resulting in different model performances. Interestingly, the corresponding model for dicyanamide shows a good correlation with experimental α (see Table T1). Similar attempts to identify more local models for π^*^ did not yield good results. Given the rather weak correlations with the response, we believe that the computed parameters are insufficient to describe the dipolarity/polarizability effects. Spange et al. (2014) had noted that π^*^ could not be adequately described as a function of the cation structure. However, we observe that for some ILs, a large β value is typically associated with a large value of π^*^ (see Figure F4).

### 3.2. Model Evaluation

Using a larger and more diverse dataset, our results suggest that the model based on quantum-derived parameters can be used to predict the hydrogen-bond basicity of a wide variety of ILs. In order to the evaluate the utility of the models, we carried out an exploratory study with a set of previously synthesized cations and anions. A set of 101 cations spanning to 14 classes (ammonium, azepanium, benzimidazolium, guanidinium, imidazolium, morpholinium, oxazolidinium, phosphonium, piperidinium, pyrazolium, pyridinium, pyrimidinium, pyrrolidinium, triazolium) were combined with 89 anions (acetate, phosphonate, phenolate, sulfate etc) to yield 8,989 ILs. For these compounds, we calculated the β using Equation (2) and additionally, the logarithmic activity coefficients at infinite dilution (lnγ^∞^) of microcrystalline cellulose (MCC) in ILs.

**Figure 3:**
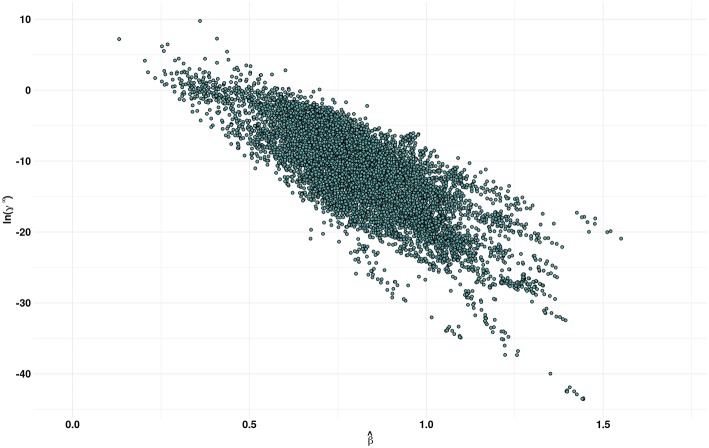
Scatter plot of $\hat{\beta }$ predicted by the MLR model and COSMO-RS estimates of the infinite dilution activity coefficients (γ^∞^) of multicrystalline cellulose in various ionic liquids.

**Figure 4:**
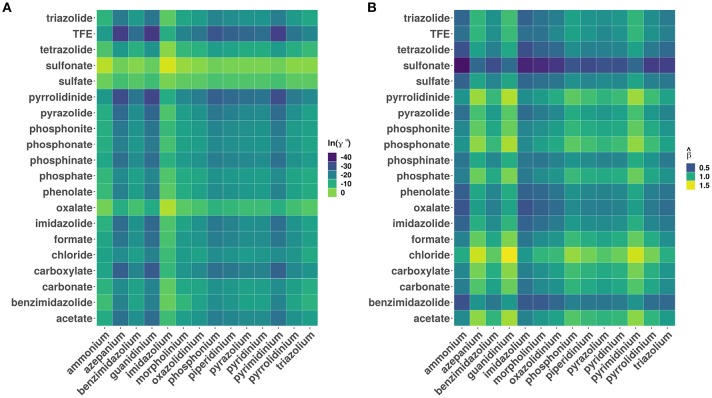
Heatmaps showing the distribution of **(A)** logarithm of infinite dilution activity coefficients (lnγ^∞^) of multicrystalline cellulose in various ionic liquids and **(B)** predicted hydrogen bond bascities ($\hat{\beta }$). The data has been grouped according to different cation and anion categories.

Figure 3 shows a plot of the predicted β values against lnγ^∞^. In general, higher the β value, higher the cellulose dissolution capacity of ILs. Brandt et al. (2013) have suggested that dissolution occurs when β ≳ 0.80 parameter. Our analysis reveals that for the ILs considered more that 50% (4671 ILs) have β > 0.8. An opposite trend was observed between the cellulose dissolution and γ^∞^ values. Among the ILs studied, chloride, acetate, phosphonate, and phosphonite anions in combination with guanidinium, azepanium, phosphonium, pyrimidinium, pyridinium and imidazolium cations have high β values (see Figure 4). An opposite trend was observed for the lnγ^∞^ values for the above mentioned ILs. In other words, ILs with higher β values show lower lnγ^∞^ values, which indicate that these ILs have higher cellulose dissolution potential. The predicted cellulose dissolution results are in line with previously reported values. For instance, 1-butyl-3-methylimidazolium chloride with a β value of 0.95 (Lungwitz et al., 2010) dissolved up to 20 wt% cellulose at elevated temperature (Swatloski et al., 2002). Similarly, 1-ethyl-3-methylimidazolium based phosphonate ILs (β > 1) dissolved more than 5 wt% of cellulose at room temperature within 3 h (Fukaya et al., 2008). Guanidinium based ILs with carboxylate anions (acetate, propionate etc) dissolved up to 10 wt% cellulose at elevated temperature (King et al., 2011). However, it must be pointed out that a high β value does not always guarantee a high cellulose dissolution potential. For instance, tetraalkylphosphonium ILs with long chain fatty acids (C2–C16) have a high H-bond basicity (>1.50) but were not able to dissolve cellulose even at very high temperatures (130°C) (Yang et al., 2014; Abe et al., 2015). This may be because of the bigger size of the anion, which prevented efficient interaction between cellulose and ILs.

## 4. Conclusions

In this study, we have carried out an evaluation of the COSMO-RS approach for predicting the hydrogen-bond acidity (α), hydrogen-bond basicity (β), and dipolarity/polarizability (π^*^) of a diverse set of ionic liquids. These properties cannot be solely described by the ability of the IL cation or anion. Using a small set of interaction energy based descriptors we have attempted to build statistical models that can lead to improved correlations with experimental data. Analysis of the model for β emphasized a strong influence of electrostatic misfit and hydrogen bond interactions, which is in line with findings by Cláudio et al. (2014). Further assessment of the model based on a comparison of the β predictions for over 8000 ILs suggested an inverse correlation with the logarithmic infinite dilution activity coefficients of multi-crystalline cellulose. Although earlier studies by Kurnia et al. (2015) suggested good correlations between α and cation-anion interaction energies for 17 NTf_2_-based ILs, we observe that such trends are not maintained for a larger set of ILs (60 ILs studied). Overall, the models computed for α and π^*^ were not sufficiently predictive and any conclusions drawn should be treated with caution.

## Data Availability

The structures of the ionic liquids, associated properties, and references are provided in the Supporting Information.

## Author Contributions

VV and KL conceived the study and performed the data collection. VV performed the calculations. Both authors wrote the paper.

### Conflict of Interest Statement

The authors declare that the research was conducted in the absence of any commercial or financial relationships that could be construed as a potential conflict of interest.
