# Supplementary material for: Establishing Predictive Models for Solvatochromic Parameters of Ionic Liquids
Source: Front Chem. 2019 Sep 3;7:605. doi: 10.3389/fchem.2019.00605 (PMC6733962; doi:10.3389/fchem.2019.00605)
Supplement: Supplementary file 1 [file Data_Sheet_1.PDF]

Supplementary Material

1 MODEL EQUATIONS

Linear regression equations for α and π^* using quantum chemical parameters computed by COSMO-RS.

$$\alpha = -0.368 - 0.046H_{MF} + 0.086H_{vdw} + 3.314V_m - 0.013E_{diel} + \epsilon \tag{E1}$$

$$\alpha_{NTf2} = 2.305 - 0.372H_{MF} + 0.109H_{vdw} + 6.335V_m + \epsilon \tag{E2}$$

$$\alpha_{N(CN)2} = 8.638 - 0.284H_{MF} + 0.122H_{vdw} + 6.655V_m + 0.056E_{diel} + \epsilon \tag{E3}$$

$$\pi^* = 0.833 + 0.008H_{vdw} - 0.074E_{ring} - 0.002E_{diel} + \epsilon \tag{E4}$$

$$\pi^*_{NTf2} = 0.239 + 0.006H_{vdw} - 0.008E_{diel} + \epsilon \tag{E5}$$

$$\pi^*_{N(CN)2} = 1.146 + 0.009H_{vdw} - 0.079E_{ring} + \epsilon \tag{E6}$$

2 SUPPLEMENTARY TABLES AND FIGURES

Property	R^2	AARD(%)	RMSE
α	0.42	28.11	0.20
α_{NTf2}	0.51	24.76	0.10
$\alpha_{N(CN)2}$	0.87	12.77	0.04
π^*	0.28	6.84	0.11
π^*_{NTf2}	0.28	5.87	0.04
$\pi^*_{N(CN)2}$	0.29	3.07	0.02

Table T1. Performances of the linear regression models for α and π^*

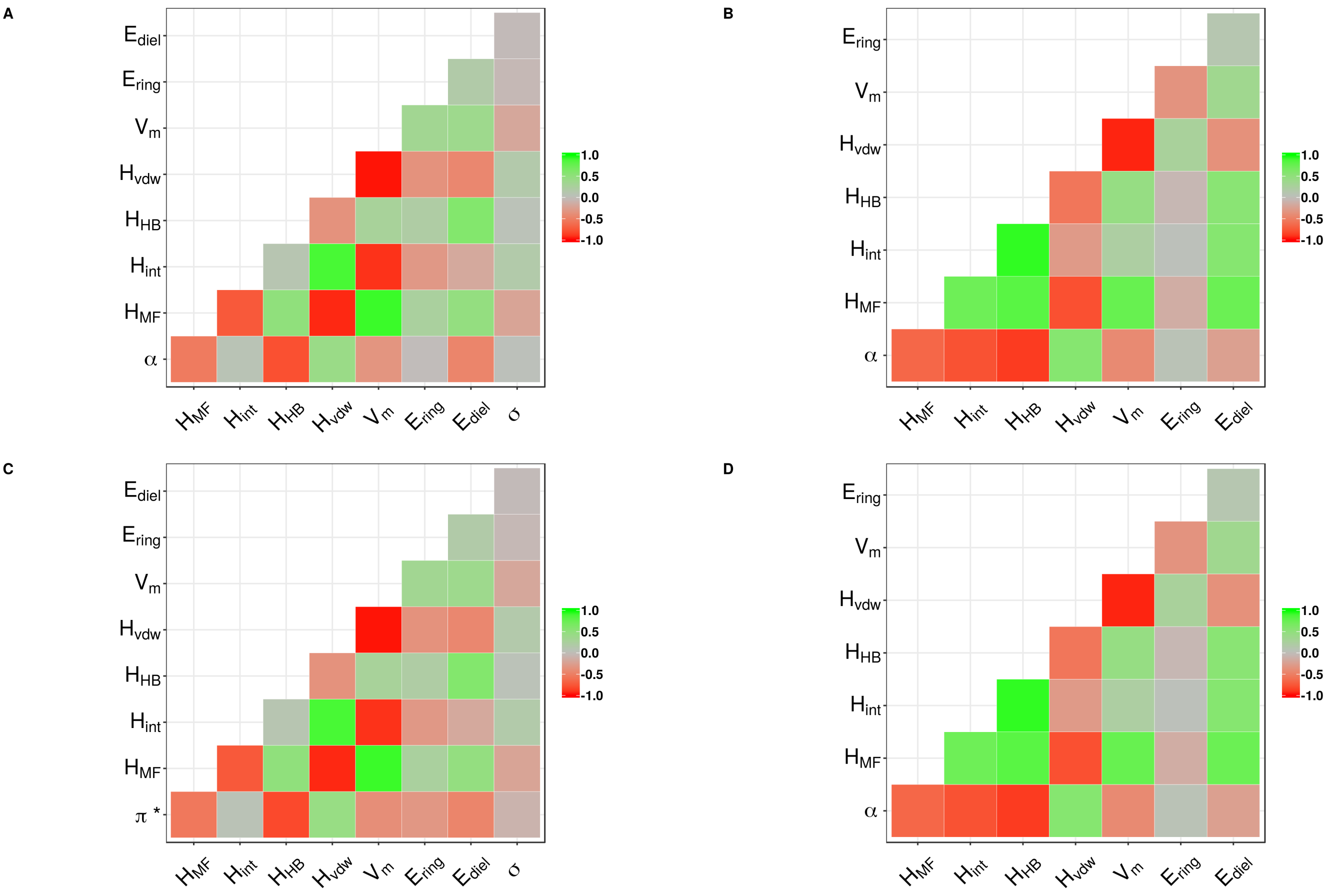

Figure F1. Correlation plots for α and π^* for ionic liquids based on (A) NTf2 (B) N(CN)2 (C) NTf2 (D) N(CN)2

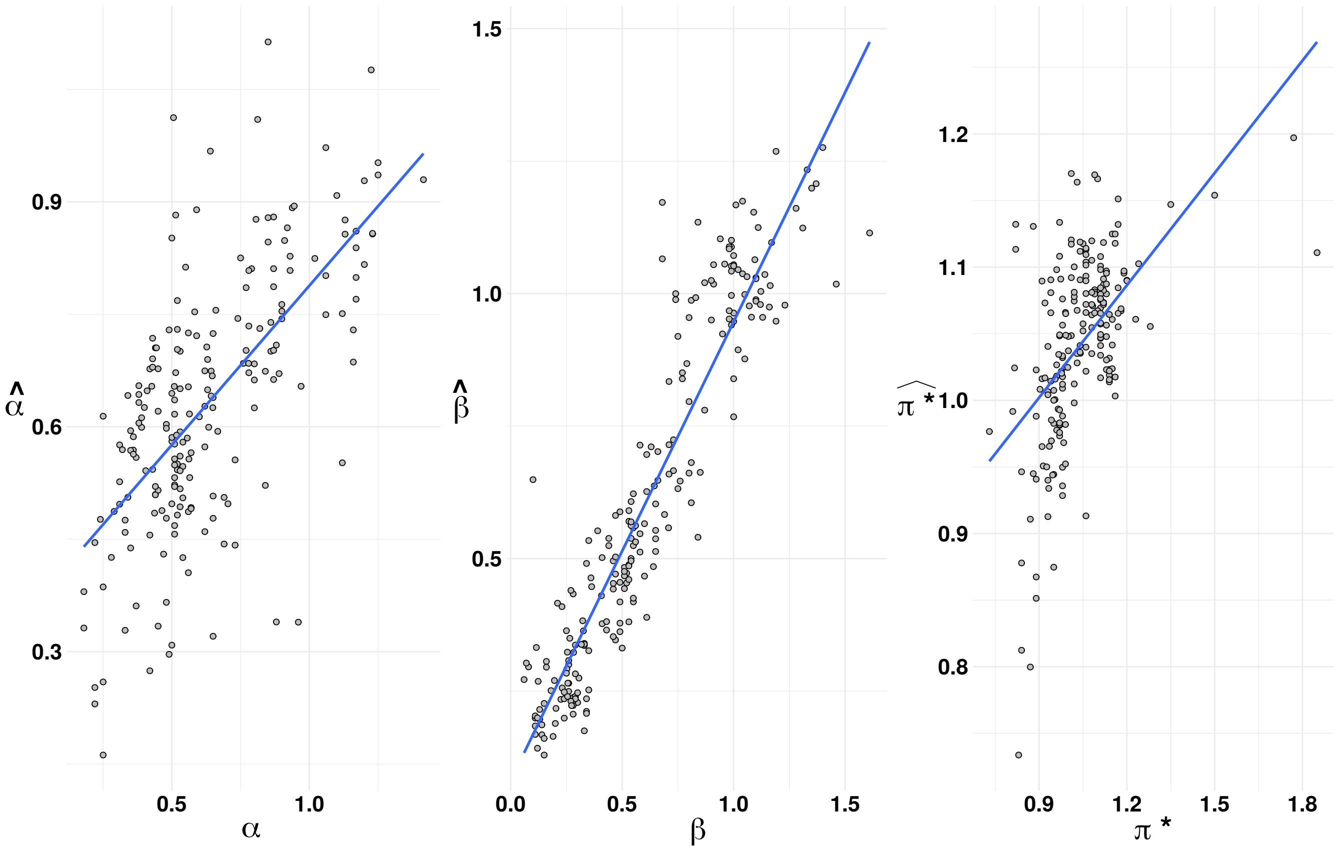

Figure F2. Experimental vs MLR-predicted values for α, β and π^*.

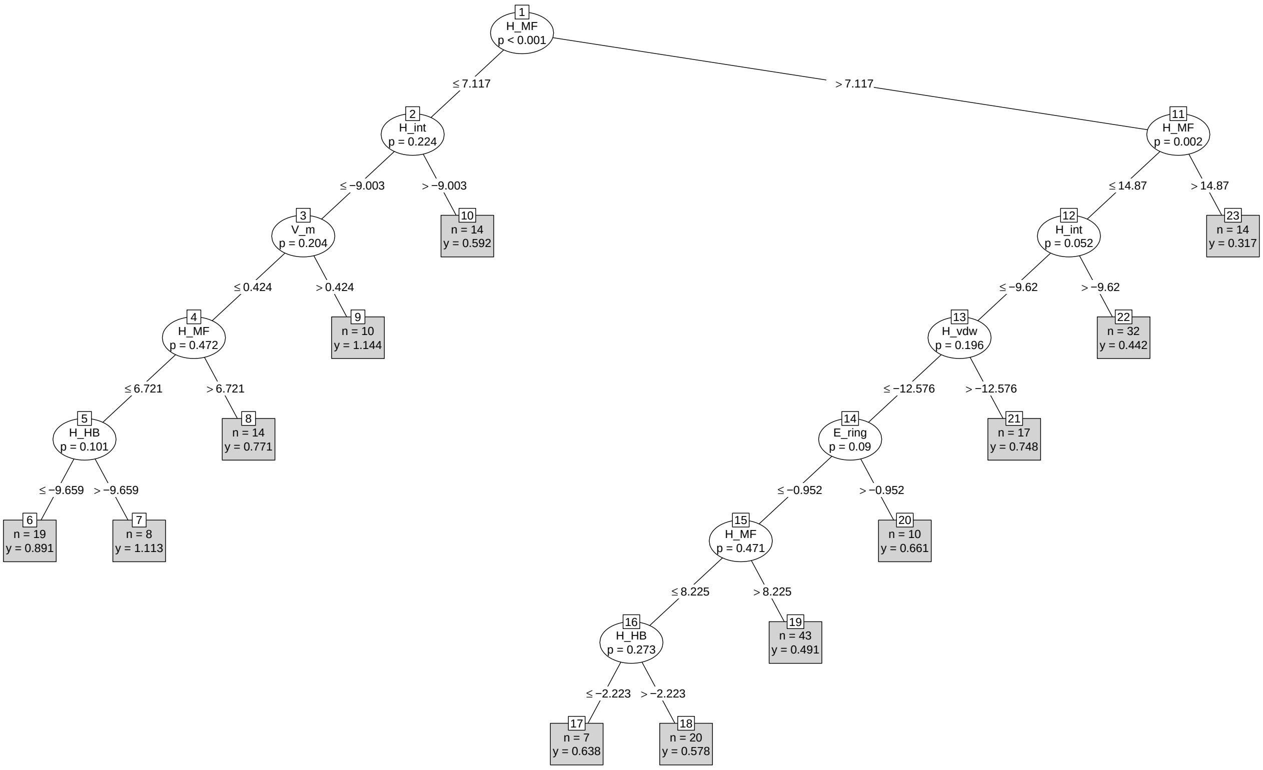

Figure F3. Decision tree model for α.

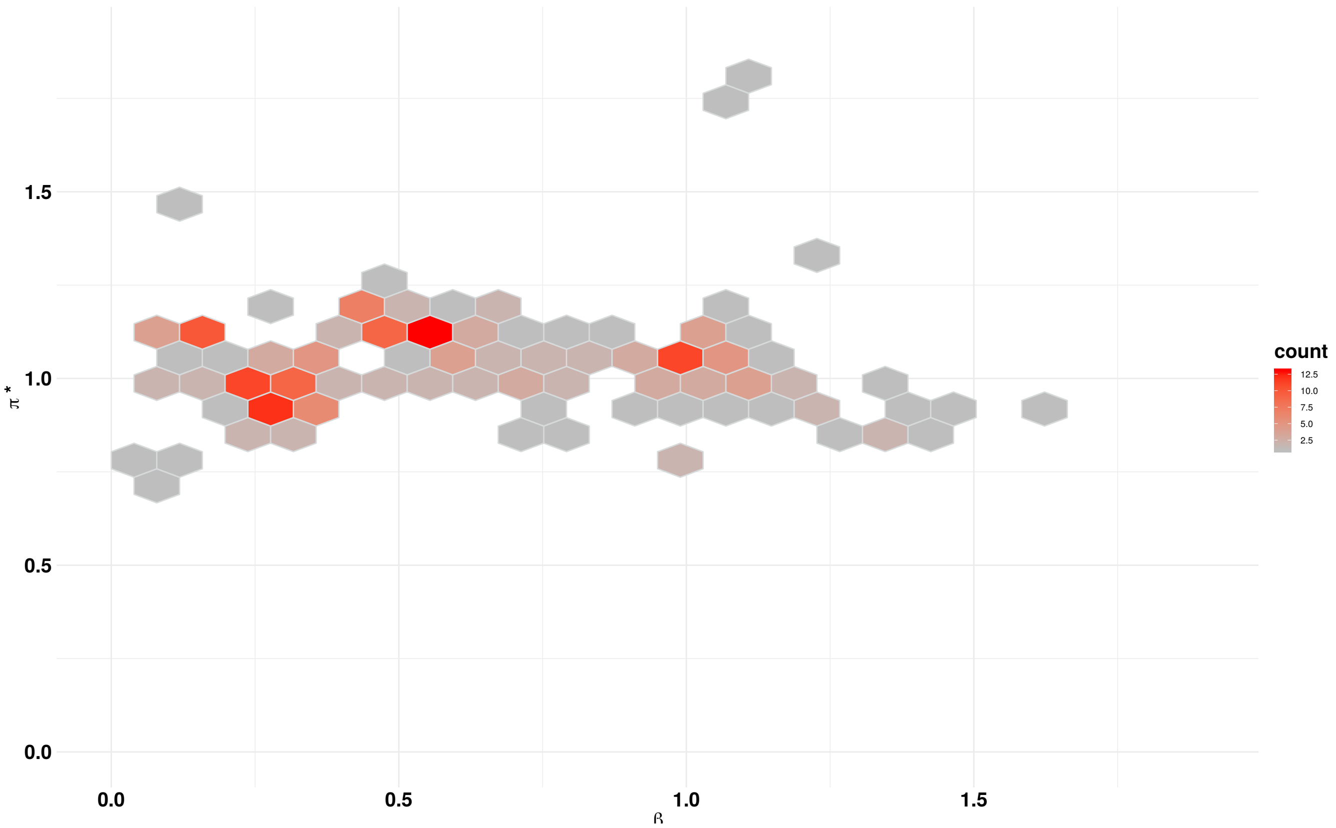

Figure F4. 2D histogram showing the counts of the occurrence of combinations of β and π^*.
